# Luminescence Probes in Bio-Applications: From Principle to Practice

**DOI:** 10.3390/bios14070333

**Published:** 2024-07-08

**Authors:** Tao Yan, Fan Weng, Yang Ming, Shijie Zhu, Miao Zhu, Chunsheng Wang, Changfa Guo, Kai Zhu

**Affiliations:** Department of Cardiovascular Surgery, Zhongshan Hospital Fudan University, Shanghai 200032, China; 19211210038@fudan.edu.cn (T.Y.); 21211210056@m.fudan.edu.cn (F.W.); 19211210048@fudan.edu.cn (Y.M.); zhu.shijie@zs-hospital.sh.cn (S.Z.); 20211210111@fudan.edu.cn (M.Z.)

**Keywords:** luminescence probe, fluorescence, photoacoustic, bio-application

## Abstract

Bioanalysis based on optical imaging has gained significant progress in the last few decades. Luminescence probes are capable of detecting, monitoring, and tracing particular biomolecules in complex biological systems to figure out the roles of these molecules in organisms. Considering the rapid development of luminescence probes for bio-applications and their promising future, we have attempted to explore the working principles and recent advances in bio-applications of luminescence probes, in the hope of helping readers gain a detailed understanding of luminescence probes developed in recent years. In this review, we first focus on the current widely used luminescence probes, including fluorescence probes, bioluminescence probes, chemiluminescence probes, afterglow probes, photoacoustic probes, and Cerenkov luminescence probes. The working principles for each type of luminescence probe are concisely described and the bio-application of the luminescence probes is summarized by category, including metal ions detection, secretion detection, imaging, and therapy.

## 1. Introduction

Bioanalysis based on optical imaging has gained significant progress in the last few decades [1]. It utilizes various kinds of optical responsive probes to visualize physiological and pathological processes in living subjects. Optical probes refer to a single molecule or an aggregation of molecules that undergo changes in photoinduced emission after interacting with the analytic samples [2]. These optical probes are capable of detecting, monitoring, and tracing particular biomolecules in complex biological systems to figure out the roles of these molecules in organisms [1,3]. Depending on the different mechanisms of the optical imaging, the optical probes can be categorized into Fluorescence (FL) probes, Bioluminescence (BL) probes, Chemiluminescence (CL) probes, Afterglow probes, Photoacoustic (PA) probes, and Cerenkov luminescence probes [4,5,6,7,8]. To date, conventional imaging techniques, such as ultrasound, computed tomography (CT), and magnetic resonance imaging (MRI), have excellent tissue penetration depth. However, they inevitably present poor temporal and spatial resolution, compromised signal-to-background (SBR) ratios, and low sensitivity. By contrast, the widespread application of optical probes in bioimaging relies on their advantages such as high sensitivity, high spatiotemporal resolution, and easy accessibility [9]. As a result, bioanalysis using responsive probes is a powerful tool for early diagnosis and the following treatment. In this review, we mainly focused on the recent advance of the optical responsive probes in the bioanalysis. We discuss the mechanism, the advantages, the limitations, and the biomedical applications of different kinds of optical probes. Lastly, we analyze the translation of these optical probes to clinical practices and the potential challenges remaining to be overcome for the development of more effective probes.

## 2. Principles of Luminescence Probes

### 2.1. FL Probes

**Figure 1:**
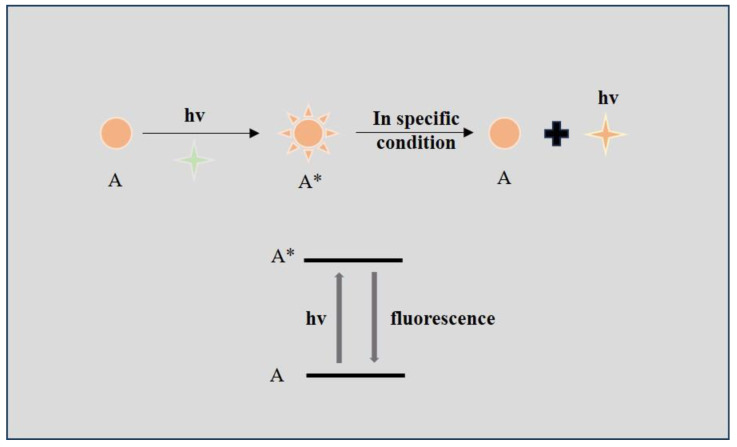
Illustration of the mechanisms of fluorescence imaging. A: substrates; A*: substrates at an excited state.

The FL probe has become a significant tool in modern biomedical analysis. It is based on the photoluminescent phenomenon and was developed to track the biochemical reactions in complicated environments [10,11]. When fluorescent substrates are exposed to external excitation light, they will release relaxed photons in the form of fluorescence and then return to the ground state, which is called the photoluminescent phenomenon [12] (Figure 1). FL probes are used by researchers to label nonfluorescent molecules in vivo and then visualize the generation, migration, and interactions of these molecules in the biological process [13,14,15]. The fluorescence microscope is used to detect the FL signal. It can block the external illumination and provide the observer with a dark background [16]. As a result, only the emitted FL signal is observed. The FL imaging offers superior spatiotemporal resolution, high sensitivity, and real-time detection at the single-molecule level [17,18]. Many kinds of FL probes have been developed for different uses, such as FL proteins, FL dyes, and nanomaterial-based probes [19]. FL proteins are mainly applied in the labeling of viruses, genes, and lipids [20,21]. FL dyes are suitable for labeling antibodies and drugs [9,22]. Newly invented nanomaterial-based probes have better stability than conventional organic probes, so they are appropriate for the long-term analysis of biological reactions [23]. The main limitations of FL imaging include the shallow tissue penetration depth and spontaneous fluorescence [24,25]. The two-photon (TP) fluorescence probes along with the near-infrared (NIR) FL probe are good tools to avoid these laminations. The TP material needs to absorb two photons to reach the excited state, so only the focused area under the TP microscope can be excited and the resolution of the image is higher [26,27,28]. Furthermore, the wavelength of the external excitation light is longer in the TP imaging, so it can penetrate deeper into the biological tissue [29,30]. The NIR light has a higher wavelength, so its penetration ability is better. As a result, the NIR FL probes are suitable for in vivo imaging [16]. For example, viscosity is a significant parameter of the micro-environment, and abnormal viscosity is usually associated with different kinds of diseases. In order to detect viscosity precisely, a NIR FL probe (QX-V) based on quinoline–xanthene dye has been developed. In the highly viscous medium, the free rotation of the single bond in the probe is inhibited and it will emit fluorescence (786 nm) after being excited by external light (710 nm) [31].

### 2.2. BL Probes

**Figure 2:**
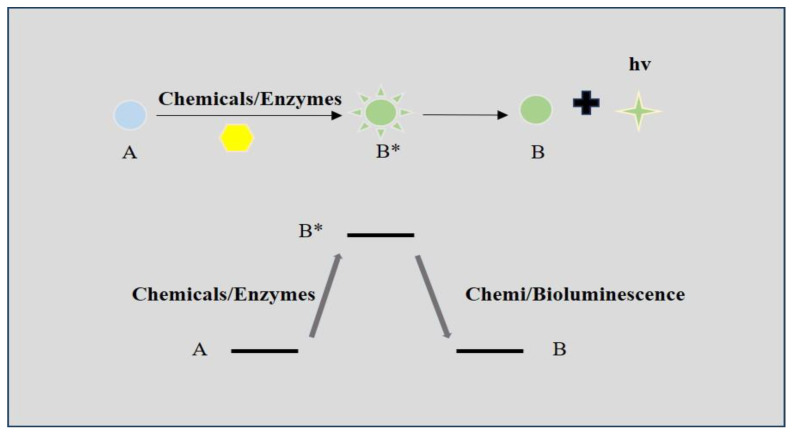
Illustrations of the mechanisms of direct chemi/bioluminescence. A: substrates; B: synthetic substrates; B*: synthetic substrates at an excited state.

A BL probe is one of the most sensitive imaging techniques in thick tissues or integrated living organisms [32]. In BL imaging, the luciferin substrates are oxidated by luciferase and generate an oxidative intermediate substrate at the excited state [33,34,35,36]. When the excited intermediate substrate relaxes to the ground state, it will emit fluorescence [37] (Figure 2). Forty kinds of BL systems have been found nowadays, but only eleven of the structure and mechanism of the luciferase–luciferin pairs have been illustrated [38]. The color and the intensity of the emitted light are determined by the different molecules of the luciferase–luciferin pairs [39,40,41]. Compared to conventional FL imaging, BL imaging (BLI) eliminates the external excitation light. As external light can lead to a fluorescent background or phototoxicity, BL imaging has higher contrast, lower toxicity, and no autofluorescence. The BLI is suitable for detecting different kinds of biochemical processes in vivo. For example, ATP plays an important role in energy exchanges in the living organism. In order to measure the concentration of ATP, the D-luciferin/62 kDa insect luciferase (Fluc) assay was developed. In the presence of ATP, the Fluc can oxidize D-luciferin to an excited state. The relaxation of the excited-state oxyluciferin species to its ground state will emit fluorescence (558 nm). The fluorescence intensity is proportional to the concentration of ATP [39]. Typical BLI releases a weak light with a wavelength shorter than 650 nm, which will easily be absorbed or scattered by the biological tissue. To solve this problem, researchers figured out two solutions. On the one hand, they tried to identify new BL probes in nature that can create brighter, stronger, and near-infrared BL signals [5,42,43]. On the other hand, they develop a new BL system to change the wavelength and stability of the emitted light by genetic engineering and chemical syntheses [44,45,46]. Nowadays, BLI is mainly applied in the measuring of pH, membrane potential, the detection of tumor growth, and high-throughput screening in the discovery of drugs [47,48].

### 2.3. CL Probes

**Figure 3:**
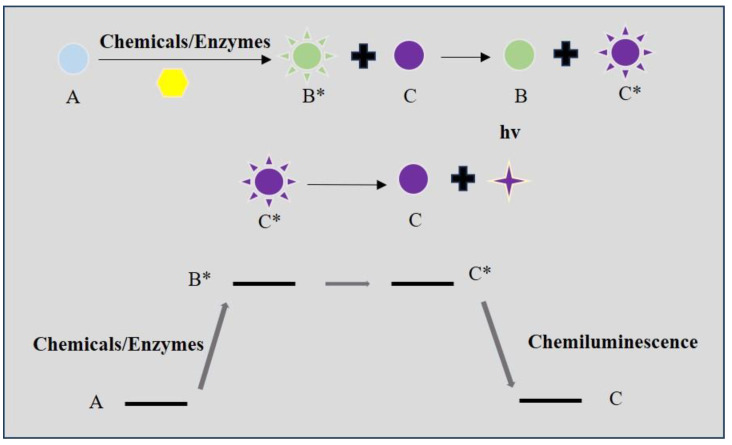
Illustrations of the mechanisms of indirect chemiluminescence. A: substrates; B: synthetic substrates; B*: synthetic substrates at an excited state; C: indirect substrates; C*: indirect substrates at an excited state.

CL imaging refers to the production of photons when electrons return from the excited state to the ground state during the chemical reaction [49,50]. Compared to BL imaging, CL imaging relies on a chemical reaction other than the oxidation [51,52]. It can be classified into two kinds based on the different chemical energy transition mechanisms [53,54,55,56]. Direct CL imaging is the process in which the substrate is transferred to an unstable synthetic substrate at the excited state and then the relaxation of the intermediate will release protons. In the indirect luminescence process, the energy of the excited intermediate is first transferred to the indirect substrates surrounding it. The photons are then emitted by the relaxation of excited indirect substrates to the ground state (Figure 3). As CL imaging eliminates external light sources, the background fluorescence in chemiluminescence is very low. Thereby, CL imaging has strong sensitivity, superior resolution, and high signal-to-noise ratios in bioimaging [57]. Many kinds of CL substrates have been developed, including 1,2-dioxetane, peroxyoxalates, luminol, and their derivatives. For example, H_2_O_2_ participates in a lot of important physiological events. Luminal can be oxidized by the H_2_O_2_ to the excited state 3-aminophthalate ion. Then, the excited state will relax to the ground state and release blue chemiluminescence (425 nm). As a result, luminol is widely applied in the detection of H_2_O_2_ concentration [53]. The CL fluorescence is detected by the charge-coupled device (CCD). The newly developed CCDs have high sensitivity and resolution. They can simultaneously detect and analyze the photon signal in microarrays and reduce the number of analytic samples [58]. CL imaging has become an effective tool for the continuous detection of physiology and pathology processes in vivo. So, CL imaging has achieved wide application in the biological analysis of proteins, enzymes, and nucleic acids [6,59,60,61]. The main limitation of the CL probe is the relatively weak signal strength [62]. Many efforts have been made to amplify the CL signal in bioimaging. Recently, the development of the triggerable dioxetane CL probe can provide bright CL signals in the physiological condition [63].

### 2.4. Afterglow Probe

**Figure 4:**
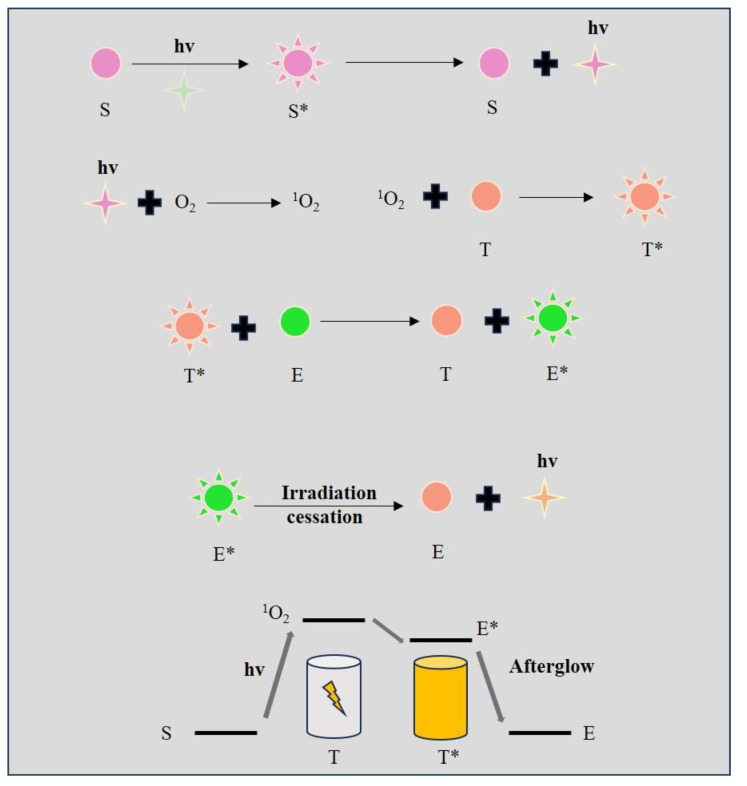
Illustration of the mechanisms of afterglow imaging. S: photosensitizer; S*: photosensitizer at an excited state; T: the trap center; T*: the trap center at an excited state; E: the emitter center; E*: the emitter center at an excited state.

Afterglow luminescence refers to the internal self-luminescence of the afterglow agent after the photoactivation [64]. Typical afterglow probes can be divided into the photosensitizer, the emitter center, and the trap center. The photosensitizer can transfer the energy of external light to oxygen and generate singlet oxygen (^1^O^2^). The trap center will react with the ^1^O^2^ and store the photoenergy. The emitter center gradually releases the energy in the form of protons [65,66,67] (Figure 4). In conventional FL imaging, the lifetime of the responsive probes is short, the imaging quality is severely influenced by the scattering of excitation light and the strong luminescence of the background [68]. In afterglow imaging, as the lifetime of the afterglow can last several seconds or even hours, the excitation light can be turned off after the afterglow agents are excited and the observer can wait for the background fluorescent to degenerate completely. As a result, afterglow imaging has several advantages including little background noise, superior sensitivity, and prolonged lifetime compared to traditional fluorescence imaging techniques [69,70,71]. The properties of the afterglow agent depend on the emitter center and the trap center [3]. The emitter center determines the wavelength of emitted light in afterglow imaging. The trap center is the significant structure that stores the photoenergy and gradually releases it in the form of photons. Therefore, the lifetime of the afterglow fluorescence relies on the trap center of the afterglow probe. The afterglow probe has been utilized as an embolic agent for real-time intraoperative visualization during transcatheter embolization (TAE). Palladium (II)1,4,8,11,15,18,22,25-octabutoxyp-thalacyanine (PdPc(OBu)8) was chosen as the photosensitizer that can transfer the energy of external light to oxygen and generate ^1^O^2^. 4-(5,6-dihydro-2-phenyl-1,4-oxathiin-3-yl)-N, N-dimethylbenzenamine played the role of trap center, which reacted with the ^1^O^2^ and stored the photoenergy. Eu (TPPO)_2_(β-NTA)_3_ served as the emitter that accepted the energy from the trap center and gradually released it in the form of photons [65]. The afterglow materials can be divided into inorganic agents and organic agents [72]. Inorganic afterglow agents usually contain metal ions that have a highly conductive structure. The application of the inorganic afterglow agent in bioimaging is limited as it will leak heavy metal ions that are toxic to the biological analytes [73]. Compared to the inorganic ones, the organic afterglow agents are more biocompatible and have high structural diversity, which is suitable for the construction of the ideal afterglow probes applied in bioimaging. However, the emission of afterglow will attenuate over time; thus, the afterglow probe has a poor quantitative capacity. Thus, the further modification of the afterglow probe’s structure and the discovery of new afterglow agents are significant for the progress of the afterglow imaging technique.

### 2.5. PA Probes

**Figure 5:**
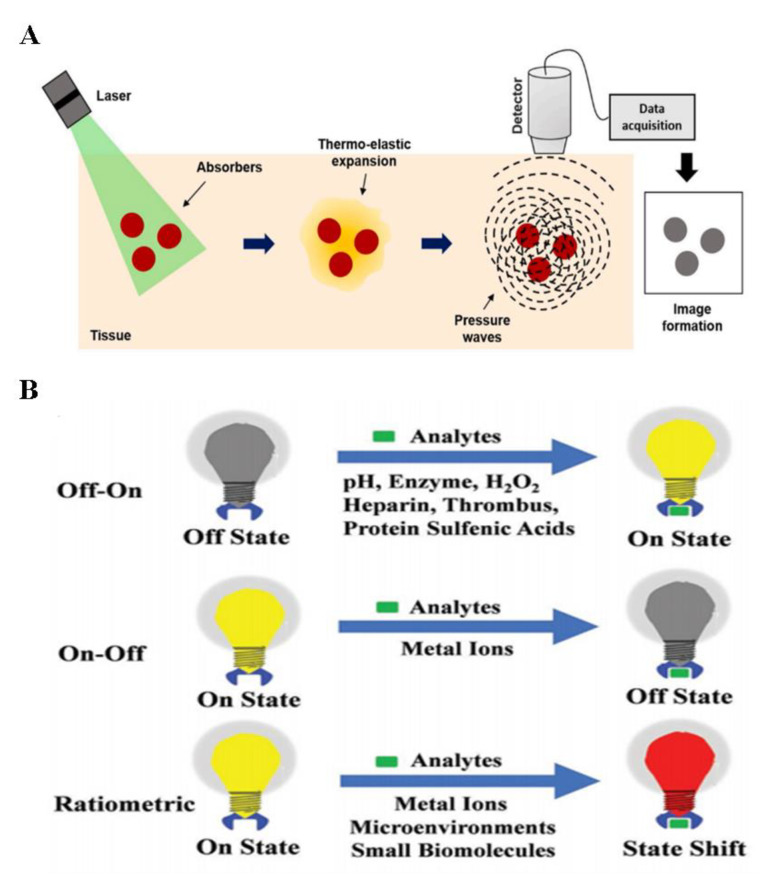
Illustrations of the mechanisms of photoacoustic imaging: (**A**) Illustrations of the mechanisms of photoacoustic imaging. Reprinted with permission from ref. [87]. Copyright 2020 Elsevier. (**B**) Illustration of different kinds of photoacoustic probes. Reprinted with permission from ref. [83]. Copyright 2018 Wiley Online Library.

The photoacoustic probe is based on the PA effect [74]. When soft biological tissues are irradiated by external light, the photons of the light will penetrate the tissues to various depths [75,76,77]. These photons are scattered and absorbed by the tissues and result in a transient increase in temperature in the local area. The transient change in temperature will lead to the thermo-elastic expansion of the tissue and generate pressure waves that propagate through the tissues in the form of ultrasonic waves (Figure 5A). These waves were recorded by ultrasonic transducers and the tissues that absorb the light were reconstructed in two or more dimensions [78,79]. PA imaging is a hybrid technique that takes advantage of both optical and ultrasound imaging. Ultrasonic waves experience less scattering and absorption during their propagation through the tissues compared to protons in the traditional optical imaging technique; thus, they can reach deeper biological tissues [80,81,82]. The construction of the PA probes can be divided into three kinds: off–on, on–off, and ratiometric probes [83] (Figure 5B). In the off–on strategy, the probe can amplify the PA signal after contacting with the analytic specimen by increasing the absorption of light. For the on–off strategy, after the probe interacts with the analytes, the absorption of the light decreases significantly and leads to the great attenuation of the PA signal. The ratiometric probe was developed to quantify the concentrations of analytic specimens in vivo. It relies on the self-calibration of the PA probes and records the changes in the PA signal caused by the analytic specimen. The main advantages of the PAI are its imaging depth and its high imaging resolution [7,84]. The PA probe is mainly used in the detection of vasculature distribution, brain functions, gene expression, tumor angiogenesis, and protein metabolism. The PA probe has been applied in the in vivo imaging of the brain. Laser pulses (532 nm) were absorbed by the skin on the head of mice. PA waves were generated and propagated through the brain tissue. These waves were then recorded by the transducers, and the distribution of optical absorption within the brain tissue was reconstructed. This technique can evaluate the changes in blood flow, oxygen consumption, and cytotoxic edema during pathophysiological events in the brain [85]. The PA probe also has several limitations. Firstly, the ultrasonic waves cannot penetrate pulmonary tissues or cavities in vivo effectively. Secondly, PA microscopes need to contact the specimen through a coupling media, which is impractical for many clinical applications [86]. Furthermore, the cost of the PA imaging technique is still very high.

### 2.6. Cerenkov Luminescence Probe

**Figure 6:**
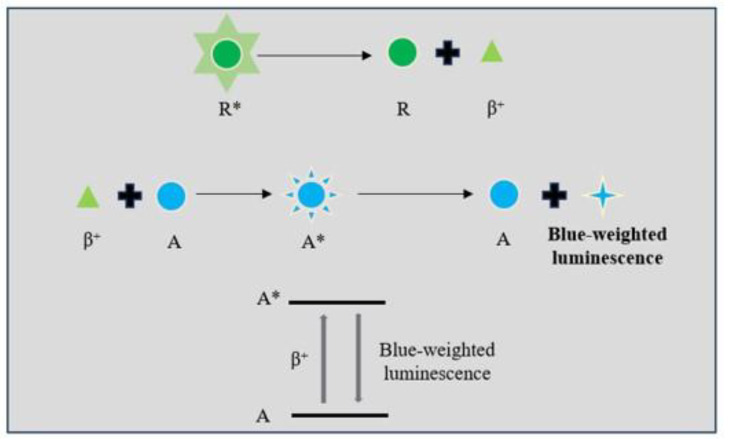
Illustration of the mechanisms of Cerenkov luminescence. A: substrates; A*: substrates at an excited state; R: stable element; R*: radioisotope.

Cerenkov luminescence refers to the blue-weighted glow emitted by charged particles (β) [8,87]. As radioisotopes are unstable, they will decay to the stable element and release energy in the form of a particle [88,89]. The majority of Cerenkov luminescence is produced by charged β particles. In water or biological tissue, charged particles travel faster than light and can result in the polarization of the dielectric substrates surrounding them. After the charged particles pass through, the remaining polarized substrates will return to the ground state and emit blue-weighted luminescence (Figure 6). This self-luminescence phenomenon is called Cerenkov radiation. The Cerenkov luminescence signal is recorded by optical cameras. CCD cameras, which have high sensitivity, were developed to provide high-resolution Cerenkov imaging. The intensity of the emitted Cerenkov luminescence signal is brightest when it is located between the UV and the visible light spectrum [90,91,92]. So, CCD cameras that can capture the fluorescence within the spectrum are well developed. However, the Cerenkov luminescence at these wavelengths is easily absorbed and scattered by the biological tissue and has limited penetration depth. Furthermore, the luminescence signal is easily disturbed by the background noise produced by β particles and γ rays. In order to overcome these shortcomings, many efforts have been made by researchers. The fluorescence resonance energy transfer (FRET) technique, which can transform the visible spectrum to a longer red or near-infrared wavelength, is a solution to the reliance of Cerenkov luminescence on visible spectrum light. Cerenkov luminescence has been applied in molecular optical imaging. As the 18F can emit high-energy charged β particles, it is widely used in Cerenkov luminescence imaging. Due to the Warburg effect, the glucose uptake is upregulated in the tumor. As a result, 2-deoxy-2-(18F) fluoro-D-glucose (18F-FDG) is selectively accumulated in the tumors after being injected into the mouse with glioma and can be used for imaging of the tumor location and metabolism [93]. To remove the background noise in the imaging, novel median filters such as a detection-based fuzzy switching median filter framework have been developed [94,95].

## 3. Bio-Applications of Luminescence Probes

### 3.1. Metal Ions and Chemical Compounds Detection

#### 3.1.1. Zinc Ions

Zinc ions play a vital role in biomedical science, participating in a multitude of physiological and pathological processes [96,97,98]. As an essential trace element, zinc is involved in enzymatic reactions, cellular signaling, immune function, and DNA replication, etc. [99,100,101]. It serves as a cofactor for numerous enzymes and transcription factors, regulating gene expression and protein synthesis [102,103]. Additionally, zinc has been implicated in the development and maintenance of the central nervous system, with studies linking zinc deficiency to neurodegenerative disorders [104,105,106].

**Figure 7:**
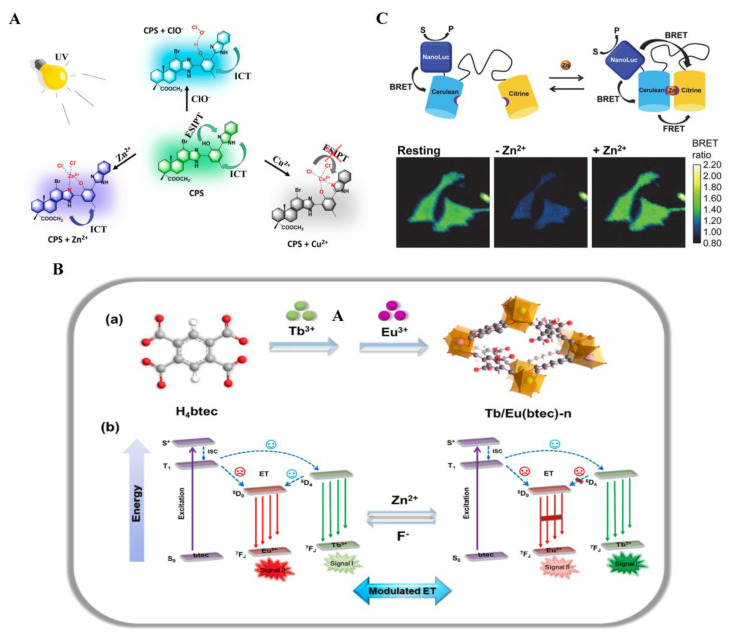
Luminescence probes for zinc ions detection: (**A**) The proposed sensing mechanisms of fluorescent probe (CPS) toward ions. The probe CPS can selectively recognize Cu^2+^, Zn^2+^, and ClO^−^ ions from other analytes by showing a ratiometric response. Reprinted with permission from ref. [116]. Copyright 2023 Royal Society of Chemistry. (**B**) The self-assembly process(a) and energy transfer pathways(b) of the Sha’s fluorescence probe. The luminous color can be finely modulated by changing the molar ratio of Tb^3+^ and Eu^3+^ during synthesis. Reprinted with permission from ref. [117]. Copyright 2023 Elsevier. (**C**) Dual readout BRET/FRET sensors developed by Aper et al. The bright and stable luciferase NanoLuc was used to create the genetically encoded BRET sensors for measuring intracellular Zn^2+^. Reprinted with permission from ref. [113]. Copyright 2016 American Chemical Society.

Luminescence sensors hold promise for real-time monitoring of zinc ions in blood serum, drinking water, or cells. The sensors utilized for monitoring Zn^2+^ are commonly classified into two categories, small molecule sensors and genetically encoded biosensors [107], which encompass various types of sensors, including single-wavelength probes [108,109,110], FRET sensors [111], and bioluminescent sensors [112,113,114]. Yang et al. synthesized a set of novel fluorescence probes called HL1-6. These probes demonstrated notable selectivity and sensitivity toward Zn^2+^ in the presence of nitrate ions, with a detection limit of 2.3 × 10^−8^ M (HL5) [115]. Sun et al. synthesized a multifunctional fluorescent probe (CPS) by incorporating the fluorophore into dehydroabietic acid. The designed probe CPS demonstrated selective recognition capabilities for Zn^2+^ in the presence of other analytes with a 0.253 μM detect limitation (Figure 7A). It exhibited notable features such as high selectivity, fast response time (15–20 s), broad pH range (3–10), and excellent photostability for ratiometric responses to Zn^2+^, manifesting a change in fluorescence color from green to blue [116]. In Sha’s study, a series of mixed lanthanide metal–organic frameworks (Ln-MOFs) probes were synthesized utilizing a facile in situ doping method (Figure 7B). By adjusting the molar ratio of Tb^3+^ and Eu^3+^ during the synthesis, the emitted color from the probes could be finely tuned. Leveraging the unique energy transfer modulation mechanism, the probes exhibited continuous detection capabilities for Zn^2+^, demonstrating the promising practical application potential of the probes. Specifically, when excited at 262 nm, the developed sensor enabled sequential detection of Zn^2+^ concentrations ranging from 10^−8^ to 10^−3^ M (with a limit of detection of 4.2 nM). Moreover, utilizing the distinct output signals, a customized device was constructed, enabling intelligent visualization for monitoring zinc ions [117].

There are few studies on bioluminescence sensors for zinc ion detection. Aper et al. developed the first genetically encoded bioluminescence resonance energy transfer biosensors for measuring intracellular Zn^2+^, called BLZinCh [113] (Figure 7C).

**Figure 8:**
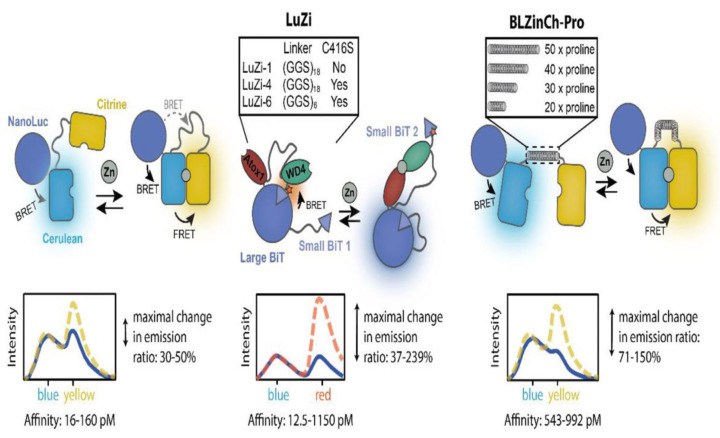
Schematic illustration of the bioluminescent Zn^2+^ sensor protein platforms. Reprinted with permission from ref. [112]. Copyright 2022 American Chemical Society.

Considering the limited response and suboptimal affinity of BLZinCh, the LuZi platform developed by Michielsen et al. employs the competitive complementation of luciferase, resulting in a quick red-to-blue emission change that enables the detection of zinc ions within the range of 2 pM to 1 nM [112] (Figure 8). They also developed an alternative platform by substituting with rigid polyproline linkers, leading to a series of advanced biosensors [112]. These sensors exhibit a three-to-four-fold enhanced response and demonstrate physiologically relevant Zn^2+^ affinities within the range of 0.5 to 1 nM. This presents an optional alternative to more labor-intensive and indirect methods for measuring serum zinc levels. Furthermore, the sensors can be genetically encoded, allowing their application as intracellular sensors. With a sensor occupancy of 40–50%, they are ideally suited for monitoring intracellular free zinc ions concentration changes in a straightforward way, eliminating the need for fluorescence microscopy.

#### 3.1.2. Sodium Ions

Sodium ions play a crucial role in various physiological processes and have significant implications in biomedicine [118,119]. As an essential electrolyte, sodium ions are involved in maintaining fluid balance, regulating osmotic pressure, and facilitating nerve impulses and muscle contractions [120]. Sodium channels, such as voltage-gated sodium channels, are vital for the propagation of action potentials in neurons and are targeted by many drugs used to treat neurological disorders [121,122,123]. Furthermore, sodium ions are intricately linked to the regulation of blood pressure and cardiovascular function. Dysregulation of sodium homeostasis has been associated with several pathological conditions, including hypertension, heart failure, and renal diseases [124,125,126].

**Figure 9:**
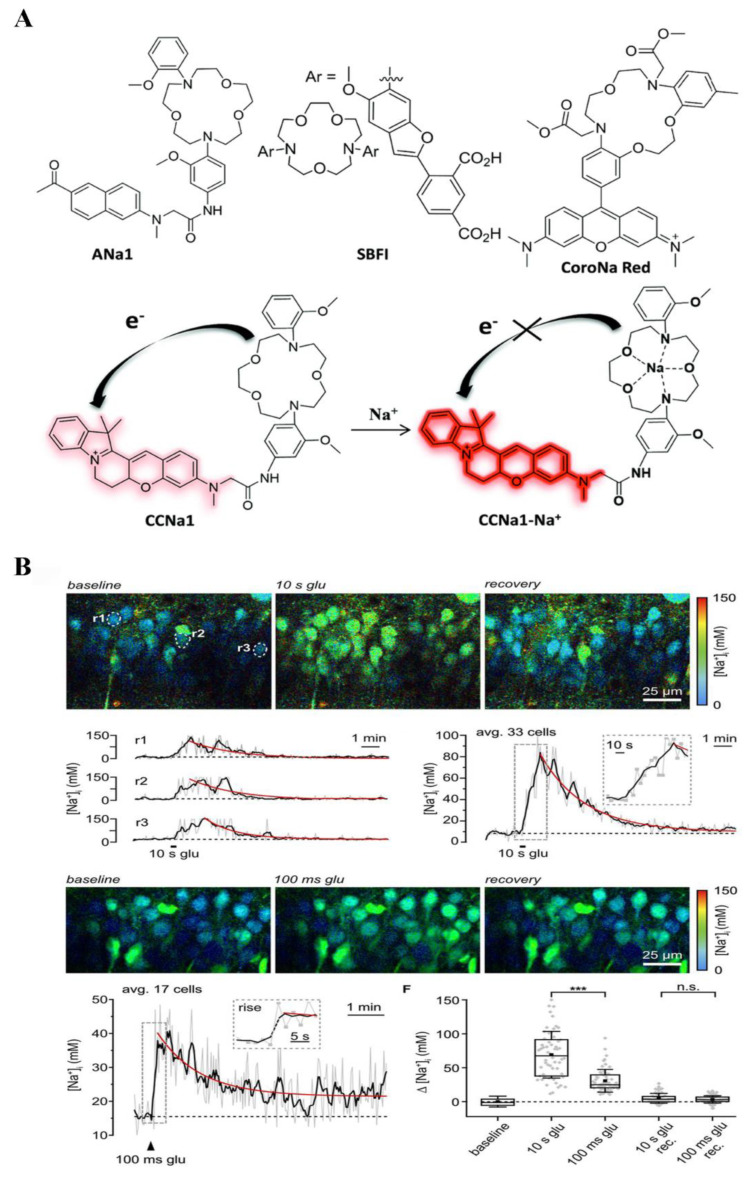
Luminescence probes for sodium ions detection: (**A**) An organic small molecule two-photon fluorescent probe (CCNa1) was developed for mitochondrial sodium ion sensing, exhibiting a low solvatochromic shift and strong fluorescence enhancement. The design consists of 1,7-diaza-15-crown-5 as the Na^+^ ion receptor and a red-emissive fluorophore based on a cyclocyanine derivative consisting of a rigid hemicyanine core. Reprinted with permission from ref. [127]. Copyright 2021 Royal Society of Chemistry. (**B**) Detection of Na^+^ signals by rapidFLIM. The rapidFLIM could reliably report both the rise as well as the decay phase of the resulting Na+ transients. Reprinted with permission from ref. [130]. Copyright 2022 Society for Neuroscience. *** *p* < 0.001.

**Figure 10:**
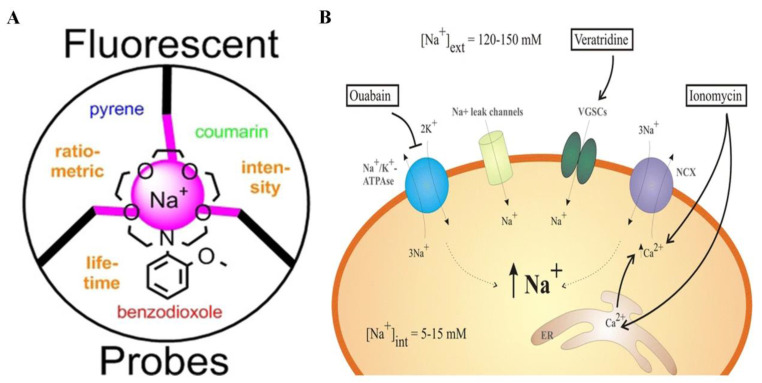
Detection of sodium ions by luminescence probes: (**A**) A set of highly Na^+^ selective fluorescent probes using click chemistry, showing Na^+^ induced fluorescence intensity or lifetime changes or a ratiometric behavior at two emission wavelengths. Reprinted with permission from ref. [128]. Copyright 2019 Wiley Online Library. (**B**) Schematic representation of the molecular pathways for intracellular sodium detection. Ouabain inhibits the activity of Na^+^/K^+^ ATPase, veratridine results in Na^+^ increase, and Ionomycin boosts the activity of Na^+^/Ca^2+^ exchanger. Reprinted with permission from ref. [129]. Copyright 2016 Springer Nature.

Juveker et al. designed a small organic molecule fluorescent probe (CCNa1) based on cyclocyanine specifically for the detection of sodium ions within mitochondria using a two-photon excitation method [127] (Figure 9A). CCNa1 exhibited a notable fluorescence enhancement at 575 nm, along with a minimal solvatochromic shift, upon binding to Na^+^. Importantly, it displayed high selectivity for Na^+^ over other metal ions and pH variations. Moreover, CCNa1 demonstrated rapid cellular uptake, biocompatibility, and remarkable sensitivity in detecting mitochondrial Na^+^ influx in live cells and mouse brain tissue. Schwarze et al. synthesized a series of fluorescent probes with different combinations of Na^+^-responsive ionophore units and fluorophore moieties [128] (Figure 10A). These probes were designed to enable fluorescence analysis based on intensity enhancements or fluorescence lifetime changes for intra- or extracellular Na^+^ detection. They developed a probe demonstrating effectiveness in measuring Na^+^ levels in blood samples based on lifetime changes. Moreover, they designed another probe to exhibit a ratiometric fluorescence response to Na^+^ at two emission wavelengths, namely, 404 nm and 492 nm. Iamshanova et al. evaluated three fluorescent dyes, namely, SBFI, Corona, and ANG-2, based on various factors, to determine the most suitable probe for visualizing Na^+^ fluctuations in vitro [129] (Figure 10B). Fluorescence imaging plays a crucial role in the analysis of various cellular and molecular processes, offering valuable insights into the detection of ions. Meyer et al. utilized fluorescence lifetime imaging (FLIM) to overcome the limitation that intensity-based approaches in fluorescence imaging are susceptible to artifacts arising from changes in fluorophore concentrations [130] (Figure 9B). Using hippocampal tissue slices from mice, loaded with the Na^+^ indicator ING2, they showcase the enhanced capabilities of rapidFLIM, a novel approach, enabling quantitative and dynamic imaging of neuronal Na^+^ signals.

#### 3.1.3. Calcium Ions

Calcium ions act as crucial signaling molecules involved in cellular communication, neurotransmission, muscle contraction, enzyme activation, and gene expression [131,132]. Disruptions in calcium homeostasis have been linked to various diseases, including neurodegenerative disorders, cardiovascular diseases, and cancer [133,134,135]. The understanding of calcium ion dynamics and its impact on cellular functions has led to the development of specialized probes and imaging techniques that allow real-time monitoring of intracellular calcium levels [136,137]. These innovative tools have greatly contributed to advancing our understanding of calcium signaling pathways and their involvement in the progression of diseases.

**Figure 11:**
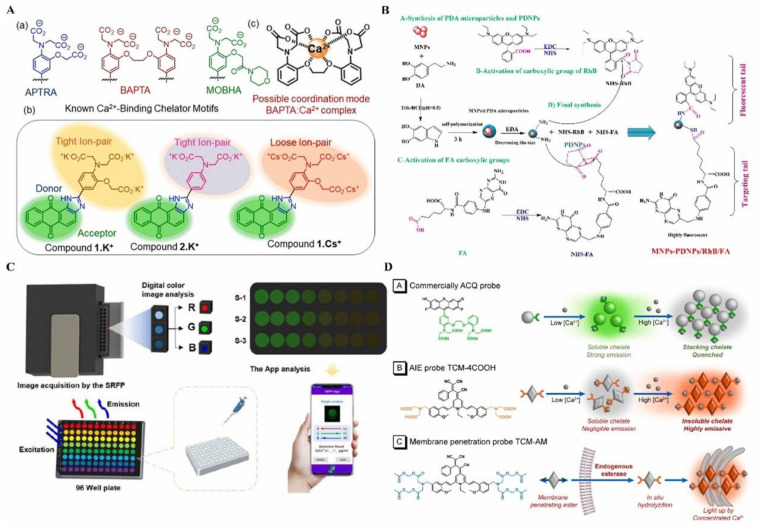
Luminescence probes for calcium ions detection: (**A**) Structures of probes for Ca^2+^ detection. Fluorogenic probes appended with a 1,2-bis-(o-aminophenoxy)ethane-N,N,N′,N′-tetraacetic acid moiety (BAPTA) are generally employed for sensing Ca^2+^ ions. The possible coordination mode for the BAPTA.Ca^2+^ complex is shown. An anthraimidazoledione-based charge transfer probe with APTRA (1.K^+^ and 1.Cs^+^) as the binding site was developed for ion detection. Reprinted with permission from ref. [138]. (a) Structures of chelating ligands known for Ca2+ ion binding. (b) Possible coordination mode for the BAPTA.Ca2+ complex. (c) Structures of compounds. Copyright 2023 Royal Society of Chemistry. (**B**) Synthesis approach of the MNPs-PDNPs/RhB/FA nanoparticles. The MNPs were dispersed in PBS buffer at pH 8.5, sonicated, and mixed with dopamine for polymerization, followed by ethylenediamine addition to create fluorescent PDNPs. RhB and FA were activated with EDC/NHS in PBS buffer and attached to the amine groups of PDNPs. Finally, MNPs-PDNPs were reacted with RhB-NHS and FA-NHS, purified by dialysis. Reprinted with permission from ref. [139]. Copyright 2022 Royal Society of Chemistry. (**C**) Schematic illustration of SRFP system for calcium detection. Reprinted with permission from ref. [141]. Copyright 2022 MDPI. (**D**) Schematic diagram of ACQ and AIE-active probe. ACQ probes exhibit strong emission in dilute aqueous solutions after binding with Ca^2+^, but their fluorescence is quenched in concentrated solutions. TCM-4COOH shows weak emission in dilute aqueous solutions, but generates bright fluorescence in aggregates after binding with Ca^2+^. The membrane-penetrable TCM-AM rapidly enters the cell membrane and is hydrolyzed by endogenous esterase into TCM-4COOH, which can be activated in calcium-rich areas. Reprinted with permission from ref. [142]. Copyright 2022 Elsevier.

Dey designed a novel amphiphilic probe based on anthraimidazoledione for the dual-mode detection of Ca^2+^ in a buffered medium [138] (Figure 11A). The compound exhibits a deep pink color, while upon the addition of Ca^2+^, the solution undergoes a color change to orange accompanied by the emergence of blue fluorescence. A noteworthy advantage of this method is its naked-eye response, eliminating the need for sophisticated visualization instruments during analysis. A magnetic-PDNP/RhB/FA nanoparticle was synthesized by Salek-Maghsoodi’s groups for the calculation of Ca^2+^ in both cell lysates and water samples [139] (Figure 11B). The resulting nanoprobe exhibited a bright emission at 576 nm when excited at 420 nm. Upon the addition of calcium ions, the fluorescence emission of the probe decreased proportionally within the concentration range of 20 ng/mL to 100 ng/mL and 0.5 μg/mL to 20 μg/mL. Importantly, this sensor demonstrated low interference in the presence of potential coexisting ions, exhibiting excellent biocompatibility and displaying favorable affinity toward FR-positive cancer cells, enabling effective bioimaging of MCF 7 cells. The portability and widespread availability of smartphones have facilitated their integration as sensors in the field of biological sciences and biomedical applications [140]. Wu’s study focused on the development of a portable smartphone-based ratiometric fluorescence probe (SRFP) platform for the rapid detection and quantification of Ca^2+^ [141] (Figure 11C). The platform utilized a cost-effective and portable setup, including a 3D-printed housing and low-cost optical components, coupled with customized software. The ratio of the green channel to the red channel in the fluorescence emissions demonstrated a consistent relationship with Ca^2+^ ion concentration. This low-cost SRFP platform holds great potential for the on-demand, rapid detection of Ca^2+^, even in remote environmental settings, enabling efficient monitoring by farmers. However, the current commercially available probes possess limitations due to their high affinity and susceptibility to aggregate-caused quenching, restricting their detection capabilities to low concentrations ranging from nM to μM. Consequently, they are unable to detect higher Ca^2+^ concentrations in situ, which fall within the μM to mM range. To solve the limitation, Li et al. have developed a novel Ca^2+^ probe named TCM-4COOH, which exhibits aggregation-induced emission activity and desirable affinity for Ca^2+^. This probe demonstrates a linear response to concentrated Ca^2+^ at mM levels [142] (Figure 11D). The rapid binding between TCM-4COOH and Ca^2+^ leads to a significant fluorescence enhancement with a high signal-to-noise ratio. Additionally, the chelates formed by TCM-4COOH have limited diffusion from cells, enabling long-term imaging capability in organisms.

#### 3.1.4. Copper Ions

As an essential micronutrient, copper ions are involved in critical enzymatic reactions, such as redox processes, oxidative stress regulation, and energy production, exhibiting diverse functions and significant implications in various biological processes [97,143,144]. Furthermore, copper ions are intricately related to many diseases, including cardiovascular diseases, cancers, and neurological diseases [104,145,146].

**Figure 12:**
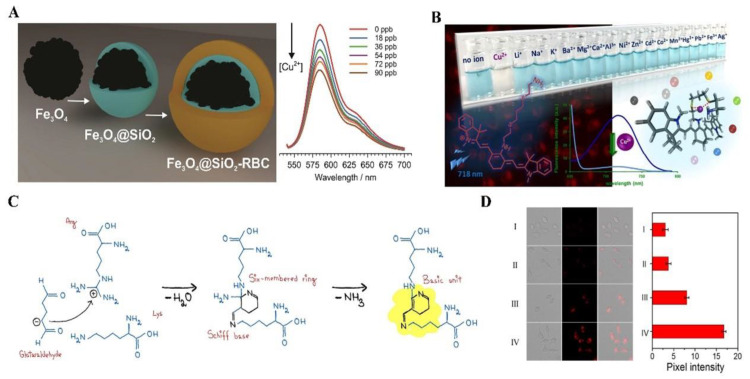
Luminescence probes for copper ions detection: (**A**) Schematic representation of the fluorescence probes. Reprinted with permission from ref. [147]. Copyright 2023 Elsevier. (**B**) Schematic representation of the Cu^2+^-selective NIR fluorescence sensor. Reprinted with permission from ref. [148]. Copyright 2020 Elsevier. (**C**) The hypothetical fluorescence mechanism of the newly developed BSA nanoparticles. It originates from the cross-linking of arginine and lysine residues by glutaraldehyde, which forms a Schiff base and a six-membered ring. This undergoes an elimination reaction, resulting in the conjugation of double bonds. The resulting chemical structure may act as a fundamental unit for the formation of large chromophores, contributing to the autofluorescence observed in BSA nanoparticles. Reprinted with permission from ref. [149]. Copyright 2023 Elsevier. (**D**) Typical CLSM images and Quantitative analysis of the SH-SY5Y cells. I-IV means various concentration of Cu2+ (I: 0, II: 50, III: 100, and IV: 200 μM). Reprinted with permission from ref. [150]. Copyright 2022 Elsevier.

Namikuchi et al. designed a fluorescence sensor based on a magnetic core–shell nanoparticle, which was functionalized with RhBCARB and utilized APTES as a linker [147] (Figure 12A). The sensor exhibits a linear response range from 10 to 90 µg/L. Importantly, the fluorescence sensor shows no interference from other metal ions. Wanichacheva’s group developed a fluorescence biosensor, named Cy7C3, based on a heptamethine cyanine dye, for the detection of copper ions [148] (Figure 12B). Cy7C3 demonstrates exceptional selectivity for Cu^2+^ ions over other competing metal ions, boasting a low detection limit of 9 ppb, which is below the maximum allowable concentration of Cu^2+^ ions in drinking water as regulated by the U.S. EPA. Notably, the naked-eye detection capability of Cy7C3 enables color change from blue to colorless, facilitating the detection of Cu^2+^. Furthermore, the sensor has been successfully applied for fluorescence imaging to detect Cu^2+^ ions in HepG2 cancer cells. Bioluminescence probes are also applied in detecting Cu^2+^. Borlan et al. presented a novel approach utilizing advanced multifunctional Glutaraldehyde cross-linked albumin nanoparticles, possessing adjustable autofluorescence emission spanning the ultraviolet-to-red spectrum with a label-free approach and low cytotoxicity [149] (Figure 12C). Mou et al. proposed a novel approach using an aptamer-functionalized DNA fluorescent sensor (AFDS) to accurately and specifically detect Cu^2+^ both in vitro and in cells, enabling sensitive detection of Cu^2+^ with an impressive detection limit of 0.1 μM and a wide linear detection range spanning from 0.1 to 300 μM. The AFDS is engineered by linking two DNA aptamers to enable a unique recognition response. By leveraging the distinct functions of each aptamer, the AFDS exhibits both tumor cell recognition capability and high-contrast detection performance. Additionally, the AFDS demonstrates remarkable specificity and selectivity in its response to Cu^2+^, effectively avoiding interference from common metal ions, chelators, and reactants. This is achieved through the irreversible interaction between nucleobases and Cu^2+^, which disrupts the topological structures and suppresses the fluorescence of the AFDS. Zhou et al. presented the design of a near-infrared fluorescent probe for the sensitive and selective quantification and visualization of Cu^2+^ in various biological samples, including living cells and brain tissues of drosophila and mice with Alzheimer’s disease [150] (Figure 12D). The application of this probe allowed a significant increase in Cu^2+^ content in the brains of disease mice and drosophila to be observed, with levels about 3.5-fold and 4-fold higher than those observed in normal mice and drosophila, respectively, helping explore the associations between Cu^2+^ and Alzheimer’s disease progress.

### 3.2. Small Molecular Detection

#### 3.2.1. Gaseous Molecule Detection

Diseases are associated with abnormal changes in the concentration of gaseous molecules such as hydrogen sulfide (H_2_S), nitric oxide (NO), and carbon monoxide (CO) [151]. These molecules participate in different kinds of pathophysiological processes, so it is of great significance to detect the concentration of them in vivo.

Kumar et al. developed a fluorescent probe, called HPB-based probe 2, based on two-photon microscopy (TPM) [152]. After adding H_2_S, the intensity of fluorescence at 380 nm is attenuated but the emission of fluorescence at 465 nm significantly increases. So, it can be used to detect the presence of H_2_S. Tang and colleagues developed a fluorescent dye called TPE-Az that is H2S-activated [153]. As a result of the quenching effect of the azido moiety presented in the TPE-Az, this probe is non-emissive. However, the H_2_S can reduce the azido moiety to the amino group and block the quenching effect. As a result, the intensity of the emitted fluorescence increases to about 60-fold after contact with H_2_S. Furthermore, when a certain threshold concentration of H_2_S is reached, we can observe a sharp increase in fluorescence intensity. The threshold is determined by the concentration of TPE-Az. So, we can detect the concentration of H_2_S by adding the sample into TPE-Az probe solutions with a concentration gradient. Chen’s group developed a NO-responsive probe called BDNA [154]. After reacting with NO, the probe’s absorption ability in the NIR-Ⅰ region enhanced gradually, and the intensity of fluorescence in the NIR-Ⅱ region increased in proportion to the NO concentration. As a result, this probe is suitable for detecting the concentration of NO in organisms. Wang and colleagues designed a probe called BTCV-CO for detecting the concentration of CO [155]. The fluorescence intensity of the probe is weak in the solution as the cis/trans isomerization of the C=C double bond can result in the decay of the singlet excited state. After incubating with CO, C=C double bond isomerization is inhibited and the fluorescent intensity increases significantly. So, this probe can be utilized for CO sensing.

#### 3.2.2. ROS Detection

Reactive oxygen species (ROS) are associated with different kinds of diseases such as cancer and inflammation. In order to sense the concentration of ROS in vivo, ROS-activated fluorescence probes have been developed. H_2_O_2_ is an important component of ROS. Murthy et al. developed nanoparticles that were composed of peroxalate and pentacene to realize the optical imaging of H_2_O_2_ in mice. The mechanism of this probe is that after adding H_2_O_2_, it will react with the peroxalate and produce an intermediate called 1,2-dioxetane. The degradation of this intermediate can generate energy. The transfer of the energy to the surrounding pentacene will result in the emission of fluorescence [156]. Many kinds of small molecules are reported to be able to be oxidized by ROS. In the presence of H_2_O_2_, 2,2′-azino-bis (3-ethylbenzothiazoline-6-sulfonic acid) (ABTS) can be oxidized to an ABTS free radical (ABTS+·), which has strong NIR absorption. As a result, a PA probe that was composed of ABTS was developed by Yan’s group to detect the concentration of H_2_O_2_. In the micro-environment, the concentration of H_2_O_2_ increased significantly. As a result, the ABTS in the PA probe was oxidized to ABTS+· and absorbed the NIR and generated a strong PA signal [157].

### 3.3. Secretion Detection

#### 3.3.1. Detection in Sweat

Sweat, a clear and watery fluid secreted by the sweat glands, contains a wide range of analytes, including electrolytes, metabolites, proteins, hormones, and drugs, which reflect the body’s health status and physiological processes [158]. The detection and analysis of sweat have gained significant importance in recent years due to its potential as a non-invasive and readily accessible biofluid for monitoring various physiological and biochemical markers, which can provide valuable insights into hydration levels, electrolyte imbalances, metabolic activity, and even the presence of certain diseases or drug use [159,160]. Furthermore, sweat-based diagnostics offer several advantages over traditional blood or urine tests, such as painless collection, real-time monitoring, and the ability to capture dynamic changes in biomarkers. Advances in wearable sensor technologies and microfluidic devices have facilitated the development of sweat-based biosensors, enabling on-site and continuous monitoring of individuals in various settings, including sports, healthcare, and occupational safety [161,162,163].

**Figure 13:**
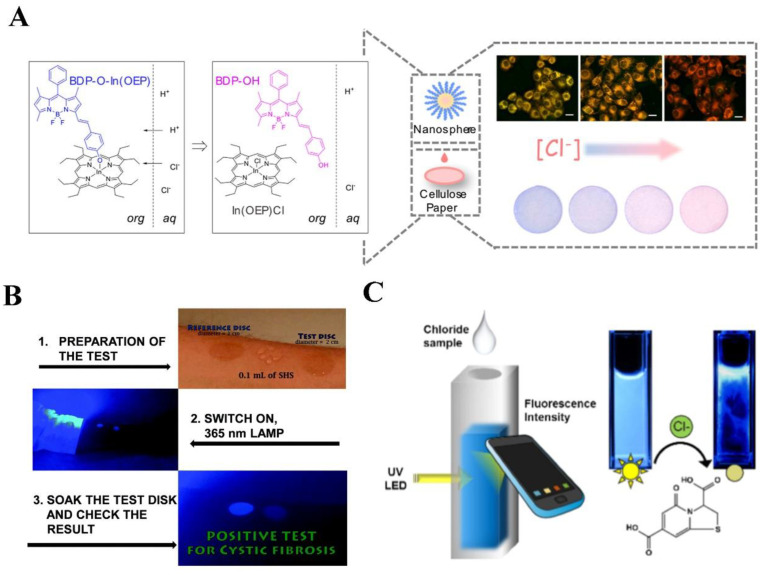
Luminescence probes for Cl^−^ detection in sweat: (**A**) Fluorescence turn-on detection of Cl^−^ with ionophore and BODIPY. The organic phase contains a chloride ionophore In(OEP)Cl and a BODIPY derivative with a phenol group (abbreviated BDP-OH). The method was evaluated in polystyrene-graft-poly(ethylene oxide) (PS-PEO) nanospheres (ca. 40 nm in diameter) and on cellulose paper. Reprinted with permission from ref. [170]. Copyright 2021 Elsevier. (**B**) Procedure for the chloride quantification in the sweat test. Reprinted with permission from ref. [171]. Copyright 2018 Royal Society of Chemistry. (**C**) A smartphone-based sensor for chloride quantification in sweat. Reprinted with permission from ref. [172]. Copyright 2017 Elsevier.

The diagnosis of cystic fibrosis (CF) in clinical practice is closely associated with fluorescence biosensors for detection in sweat [164,165,166]. CF, an autosomal recessive disease affecting the exocrine glands, is typically confirmed by quantitatively assessing chloride concentration in sweat, which serves as the gold standard [167]. However, traditional methods, such as the Macroduct collection system, which was commonly used for sweat collection in infants, have the drawback of not providing a sufficient quantity of sweat for subsequent biochemical analysis [168,169]. There have been several studies using fluorescent probes for the determination of chloride ions in sweat. Quinoline and acridine were reported as Cl^−^ Cl-sensitive fluorescence probes with the limitation of high photo-toxicity and being quenched by halides. Yang et al. proposed a biosensor using BDP-OH in the family of boron-dipyrromethene (BODIPY), displaying an increase in fluorescence upon protonation, making it an optode that responds to Cl^−^ ions [170] (Figure 13A). Cellulose papers with nanospheres, which undergo a noticeable color change in the presence of different Cl^−^ concentrations ranging from 1 mM to 1 M, were applied for monitoring Cl^−^ concentration in sweat. To detect and quantify Cl^−^ in human sweat directly on the skin, Vallejos et al. presented a novel hydrophilic polymeric film incorporating chemically anchored 6-methoxyquinoline groups as fluorescent motifs that respond to Cl^−^ ions [171] (Figure 13B). Initially, the material exhibits high fluorescence, which diminishes in the presence of Cl^−^, allowing for the quantification of Cl^−^ concentration through color analysis of a digital image or using a fluorimeter. Smartphones have emerged as powerful tools with significant implications in various fields based on their convenient accessibility and data analysis ability. Zhang et al. introduced a citrate-derived synthesis platform based on a citric acid and L-cysteine reaction to detect Cl^−^ concentration in human sweat [172] (Figure 13C). A smartphone-based chloridometer loaded with sensors was then developed to optimize the performance of Cl^−^ detection. The smartphone-operated fluorometer captured measurable changes in fluorescence emission corresponding to sweat chloride levels, exhibiting a wide linear range of 0.8–200 mM Cl^−^, demonstrating a fast response time limited by diffusion and convenient and reliable sweat diagnostics for CF patients.

**Figure 14:**
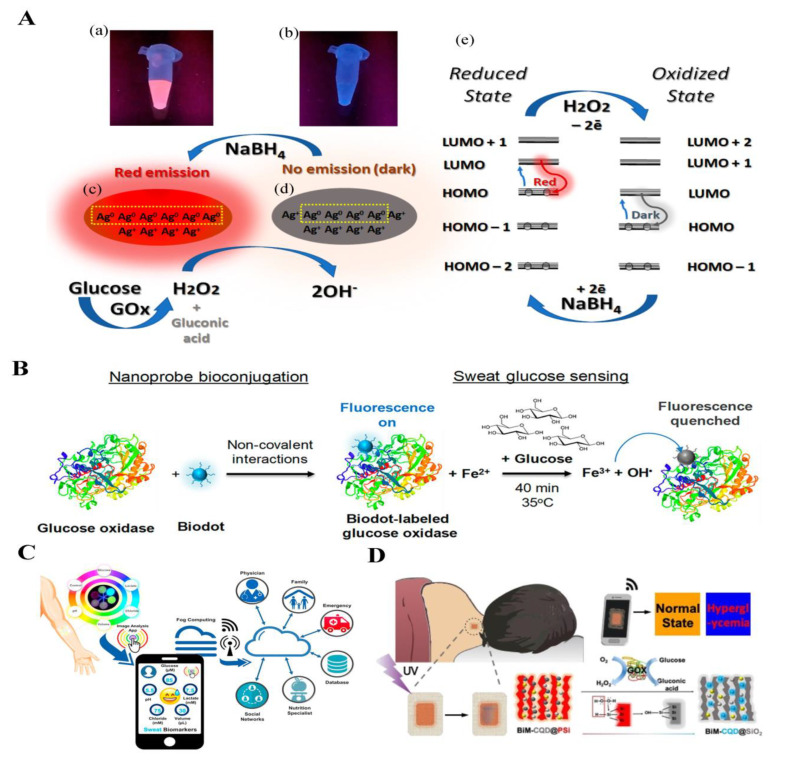
Luminescence probes for glucose detection in sweat: (**A**) Schematic illustration of dual-mode sensitive probes. Reprinted with permission from ref. [174]. Copyright 2023 MDPI. (**B**) Schematic illustration of fluorescent biodot–protein conjugates. Biodots are a new class of carbon dots derived from biomolecules or natural sources. Biodot–protein conjugate is formed via one-step noncovalent binding interactions. (a) Fluorescence of the sample excited by UV on a transilluminator. (b) Fluorescence of the sample excited by UV on a transilluminator after addition of excess H2O2. (c) The structure and composition before oxidation. (d) The structure and composition after oxidation. (e) The effect of oxidation–reduction on the redistribution of electrons. Reprinted with permission from ref. [175]. Copyright 2020 American Chemical Society. (**C**) An IoT-integrated cellulose-based microfluidic wearable patch for smartphone fluorimetric multi-sensing of sweat biomarkers. Reprinted with permission from ref. [176]. Copyright 2020 Elsevier. (**D**) Schematic illustration of fluorescent nanohybrid for sweat glucose monitoring. Carbon quantum dots (CQD) are decorated on the porous structure of luminescent porous silicon (PSi) particles. To enhance the detection sensitivity, bimetallic nanoparticles (BiM) including gold nanoparticles (AuNPs) and silver nanoparticles (AgNPs) are co-modified on PSi. Reprinted with permission from ref. [177]. Copyright 2020 American Chemical Society.

Although previous optical biosensors for glucose in sweat have focused on colorimetric methods based on glucose oxidase reaction [173], some recent studies have also explored the application of fluorescent sensors in sweat glucose monitoring. Gupta et al. presented a novel approach for the development of a self-powered biosensor capable of dual-mode detection using DNA-templated silver nanoclusters (AgNCs@DNA) [174] (Figure 14A). The small size of AgNCs@DNA offers distinct advantages as an optical probe, making it an ideal candidate for advanced biosensing applications. The fluorescence emitted by the sensor served as a readout signal, responding to the increased generation of H_2_O_2_ by glucose oxidase in the presence of elevated glucose levels. The biosensor demonstrated remarkable sensitivity with low-level limits of detection of approximately 23 μM for optical readout, surpassing the typical glucose concentrations found in sweat. Zheng et al. proposed an innovative method for the noncovalent conjugation of fluorescence nanodots to functional proteins, offering a simplified and efficient route for biosensor development [175] (Figure 14B). The presence of Fe^2+^ then leads to significant fluorescence quenching of biodots on the conjugates. The developed probe achieves a wide linear range of 25–1000 μM with a low detection limit. In situ detection of glucose in sweat by wearable devices is another promising development. Ardalan et al. introduced a wearable sweat patch based on highly fluorescent sensing probes and microfluidic channels, capable of in situ measurement of sweat glucose concentrations as well as chloride, lactate, volume, and pH values in sweat with the assistance of a smartphone [176] (Figure 14C). Cui et al. presented a novel wearable skin pad utilizing a fluorescence biosensor for non-invasive monitoring of sweat glucose [177] (Figure 14D). The biosensor consists of luminescent porous silicon particles with a porous structure and oxidation-responsive photoluminescence decay, loaded with carbon quantum dots to create a dual fluorescence system. Through efficient fluorescence resonance energy transfer (FRET), the biosensor initially exhibits red fluorescence while the oxidation induced by hydrogen peroxide weakens the FRET effect, leading to a transition in the ratiometric fluorescence from red to blue. To fabricate the wearable skin pad, the biosensor and glucose oxidase are co-immobilized in a transparent and biocompatible chitosan film, supported by an adhesive polyurethane membrane. By establishing a strong association between the ratio of fluorescence change and sweat glucose levels, clinical tests involving diabetics and healthy volunteers demonstrate the clear identification of hyperglycemia.

Fluorescence probes are also utilized for the determination of other physical and chemical properties in sweat. L-lactate in sweat is a vital metabolite to determine the physical condition and indicate specific diseases [178]. Jia et al. achieved Eu-NDC, a lanthanide metal–organic framework, using the hydrothermal method and employing 1,4-H2NDC as the ligand and Eu as the central metal, which exhibited a rapid ratiometric response to L-lactate [179]. Because the fluorescence transitioned from red to blue as the lactate concentration increased, it can serve as a fluorescent sensor for detecting L-lactate in sweat. The sensor demonstrated excellent fluorescence stability in the presence of potential interfering components commonly found in human sweat, and it displayed low detection limits for lactate in artificial sweat. A visualized molecular logic gate served as a tool for monitoring sweat lactate levels and has the potential to indicate hypoxia during exercise. It was reported that nonanal could be an attractive respiratory marker for the COVID-19 screening test [180,181]. However, respiratory analysis may be at risk of viral infection during screening. Thaveesangsakulthai et al. utilized helicene dye-encapsulated ethyl cellulose, giving the merit of specific detection of nonanal, to analyze a total of 140 sweat samples obtained from volunteers’ foreheads [182]. Notably, COVID-19-positive droplets displayed a distinct yellow fluorescence emission while COVID-19-negative patients exhibited fluorescence darkness. By setting the optimal color intensity threshold at more than 73 for positive results, the screening performance demonstrated a remarkable sensitivity of 96% and specificity of 93%, offering a rapid testing time of less than 15 min.

#### 3.3.2. Detection in Tears

Similar to sweat, tears, the clear fluid secreted by the lacrimal glands in the eyes, is also a vital indicator to monitor the physical state [183]. Despite their primary function of lubricating and protecting the ocular surface, tears have been recognized as a valuable and non-invasive source of biological information. Tears contain a wide range of molecules, including proteins, lipids, metabolites, and nucleic acids, which can serve as biomarkers for various diseases. Tears analysis provides a non-invasive and relatively simple method for diagnosing ocular and systemic conditions [184,185,186,187].

**Figure 15:**
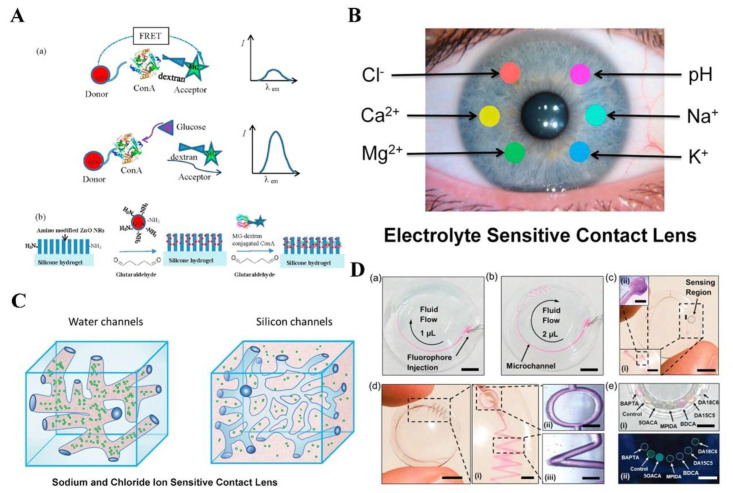
Luminescence probes for tears detection: (**A**) Schematic illustration of mechanism of the nanostructured biosensor. Reprinted with permission from ref. [192]. Copyright 2017 Elsevier. (**B**) Schematic illustration of contact lens to measure individual ion concentrations in tears. Reprinted with permission from ref. [195]. Copyright 2018 Elsevier. (**C**) Sodium and chloride ion sensitive contact lens. Reprinted with permission from ref. [196]. Copyright 2020 Elsevier. (**D**) Schematic illustration of the lens sensor for electrolyte analysis in tears. Reprinted with permission from ref. [198]. Copyright 2020 Elsevier.

Glucose in tears is considered as one of the biomarkers for diagnosis of diabetes. Badugu et al. first provided a brief overview of boronic acid-based fluorophores that have been synthesized and designed to detect glucose in tears in disposable plastic contact lenses [188,189]. The same team then presented a methodology for potential tear glucose monitoring using commercially available glucose-sensitive silicone hydrogel (SiHG) contact lenses [190]. Initially, the presence of an interpenetrating polymer network, comprising silicone and water regions, within the SiHGs was assessed using the polarity-sensitive probe Prodan. Subsequently, a glucose-sensitive fluorophore, Quin-C18, incorporating a hydrophobic side chain for localization at the interfacial region, was synthesized. Various concentrations of glucose in an in vitro system were measured through utilizing the glucose-sensing contact lens. Remarkably, the Quin-C18 exhibited strong binding to the lenses, with minimal leaching even after multiple rinses. Furthermore, the lenses maintained a consistent response to glucose even after three months of storage in water. Recent studies have utilized the fluorescence resonance energy transfer (FRET) of monitoring glucose levels in tears in order to address the response to low glucose concentrations in tears [191,192,193]. Chen et al. developed a nanostructured biosensor utilizing FRET for the detection of glucose in tears. The biosensor employs a designed FRET pair, consisting of CdSe/ZnS quantum dots (QDs) as the donor and dextran-binding malachite green (MG-dextran) as the acceptor, which are conjugated to concanavalin A (Con A) (Figure 15A). When glucose is present, the emission of QDs, which is quenched through the FRET, is restored by displacing the dextran from Con A. This dual-modulation sensor allows the conversion of glucose concentration to fluorescence spectra with a high signal-to-noise ratio and calibrated image pixel value. The photoluminescence intensity of the patterned FRET sensor exhibits a linear increase with the rising concentration of glucose within the range of 0.03 mmol/L to 3 mmol/L, covering the range of tear glucose levels observed in both diabetics and healthy individuals. Moreover, the calibrated values of pixel intensities in the fluorescence images captured by a handheld fluorescence microscope also increase with increasing glucose concentration.

In addition to glucose, electrolyte ions in tears and pH values of tears, which are associated with a number of diseases, are also a common analyte in tears detection. Ruiz-Ederra et al. introduced a novel in situ fluorescence method to measure ionic concentrations and pH values in tears [194]. They utilized dual-wavelength fluorescent indicators capable of sensing ions including Na^+^, K^+^, and Cl^−^, and pH values. Badugu et al. presented a novel method to develop SiHG contact lenses to detect pH values and chloride ions [195] (Figure 15B). They modified water-soluble fluorescent probes for pH and chloride by incorporating hydrophobic C18 chains. The resulting hydrophobic ion-sensitive fluorophores exhibited strong binding to SiHG lenses, ensuring their stability and preventing leaching when exposed to aqueous solutions. Bagudu’s group then expanded on their findings [196] (Figure 15C). They utilized Na^+^- and Cl^−^-sensitive fluorophores to noncovalently bind to two commercially available SiHGs to detect Na^+^ and Cl^−^ concentrations in tears. Yetisen et al. introduced the smartphone-based portable microfluidic system and scleral lens sensor to detect Na^+^, K^+^, Ca^2+^, Mg^2+^, and Zn^2+^ ions in tears as well as pH values of tears [197,198] (Figure 15D).

#### 3.3.3. Exhalation Products Detection

Detecting exhalation products, such as breath compounds and gases, provides a non-invasive and convenient method for monitoring the physiological and pathological conditions of an individual [199,200]. Breath analysis has shown promising potential for early disease detection and monitoring, including respiratory diseases, such as asthma, COVID-19, and chronic obstructive pulmonary disease (COPD), as well as systemic conditions like diabetes, lung cancer, and gastrointestinal disorders [201,202,203,204,205]. It allows for rapid and cost-effective screening, reducing the need for invasive procedures and improving patient comfort.

**Figure 16:**
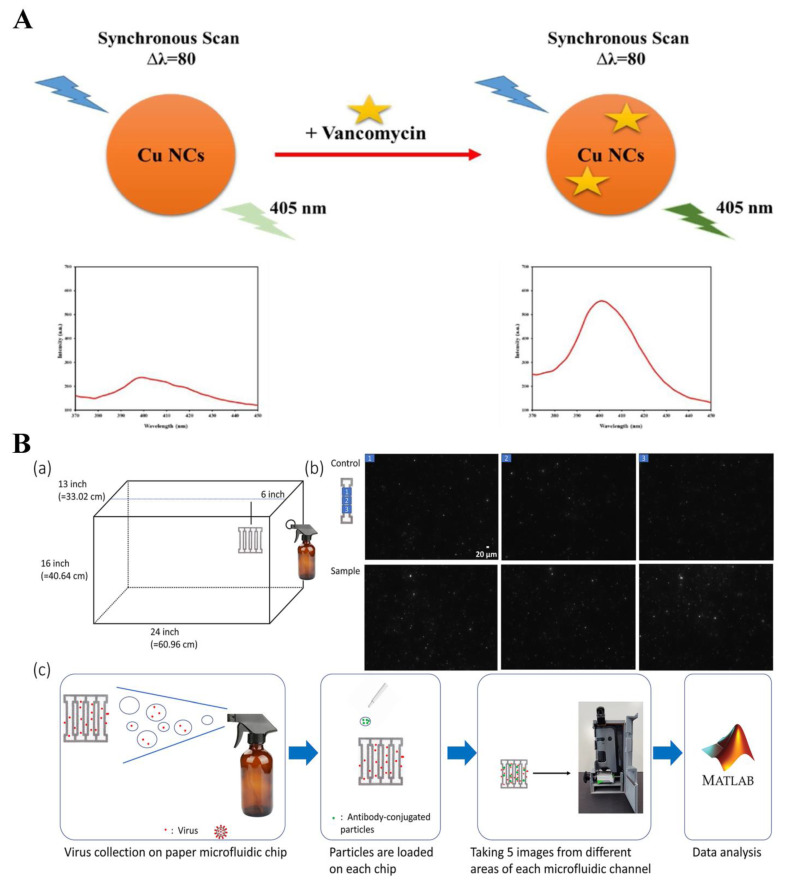
Luminescence probes for exhalation products detection: (**A**) Schematic representation of copper nanocrystal’s synchronous fluorescence spectroscopy response to vancomycin. The synthesized nanoprobe is copper nanoclusters (Cu NCs) and the affinity of Cu NCs to complex formation with vancomycin results in enhancing the synchronous fluorescence spectroscopy signal intensity. Reprinted with permission from ref. [208]. Copyright 2021 Elsevier. (**B**) Procedures of direct capture and smartphone quantification of airborne SARS-CoV-2 on a paper microfluidic chip. (a) Chamber design; (b) Fluorescence images of a paper microfluidic channel. Three different images are shown. (c) The assay procedure. Reprinted with permission from ref. [210]. Copyright 2022 Elsevier.

It was reported that breath products can be used as a non-invasive biomarker to monitor drug concentrations. Mokhtari et al. validated the use of luminol–terbium coordination polymer nanoparticles (luminol-Tb CP NPs) as a fluorometric probe for quantifying phenobarbital in the exhaled breath condensate. The probe relies on the coordination of phenobarbital with luminol-Tb CP NPs, resulting in the aggregation-induced fluorescence enhancement of the probe. By measuring the intensity of the fluorescence response, the biosensor can determine the amount of phenobarbital [206]. Mohammadzadeh et al. presented a novel strategy for detecting methotrexate (MTX) using terbium-doped dendritic silica particles (Tb@KCC-1) by leveraging the quenching effect of MTX on the fluorescence intensity [207]. The fluorescence intensity of Tb@KCC-1 is effectively quenched by MTX at 546 nm when excited at 233 nm. The degree of fluorescence quenching is directly proportional to the concentration of MTX in exhaled breath product samples. Other drugs, such as carbamazepine and vancomycin, in exhaled breath condensate could also be detected utilizing developed biosensors [208,209] (Figure 16A).

Exhaled breath products can also be detected using fluorescence probes as disease markers. Kim et al. developed a paper microfluidic chip based on smartphone quantification to detect viruses in aerosols [210] (Figure 16B).

**Figure 17:**
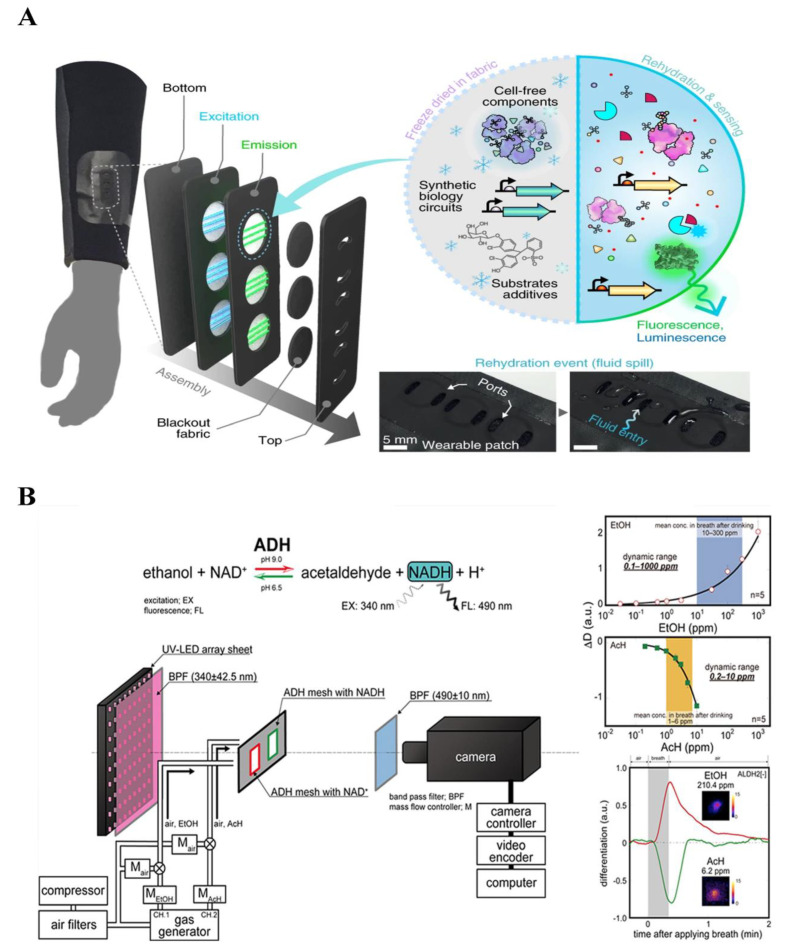
Luminescence systems for exhalation products detection: (**A**) Design of fluorescent and luminescent FDCF synthetic biology wearables. Fiber optic-embedded textiles enable the excitation and emission detection of rehydrated lyophilized biosensors. the sample wicks through the entry ports with blackout fabrics. Reprinted with permission from ref. [211]. Copyright 2021 Springer Nature. (**B**) Schematic illustration of the gas-imaging system. The imaging principle for gaseous EtOH and AcH based on the switchable redox reaction of NADH-dependent ADH is shown. Reprinted with permission from ref. [213]. Copyright 2019 Elsevier.

Nguyen introduced a novel approach to develop fluorescence devices with fiber optic detection for enhanced biosensing [211] (Figure 17A). They integrated hydrophilic threads, skin-safe patterns, and polymeric optic fibers into a platform. A customized spectrometer was utilized to visualize the readout, which could be monitored using the smartphone. They then integrated this developed biosensing platform into a medical mask for the purpose of real-time monitoring of SARS-CoV-2 in breath products. Acetone in breath products is usually detected to monitor for ketosis. Fan et al. presented a portable smartphone device, utilizing 3D-printing technology, for the primary diagnosis of diseases by detecting acetone [212]. The device incorporates red carbon dots (RCDs) as internal standards and a sensing reagent specifically designed for acetone detection. By effectively capturing acetone, the reagent undergoes a condensation reaction in an aqueous solution, forming a nonfluorescent acylhydrazone, inducing prominent color changes from blue-violet to dark red. Several fluorescence biosensing platforms have also been developed for the quantitative analysis of ethanol and isopropanol in exhaled breath products [213,214] (Figure 16B).

### 3.4. Imaging and Therapy

In recent years, luminescence probes have emerged as valuable tools in the field of in vivo imaging and targeted therapies. These probes exhibit unique luminescence properties that enable non-invasive visualization and monitoring of biological processes at the molecular and cellular levels. By selectively targeting specific biomarkers or cellular components, luminescence probes facilitate the precise localization and tracking of disease-related targets within living organisms. Additionally, their compatibility with various imaging modalities allows for multimodal imaging approaches, enhancing the accuracy and sensitivity of diagnostic assessments [79,215,216]. Moreover, luminescence probes can be coupled with therapeutic agents to create theranostic platforms, enabling simultaneous imaging and targeted treatment of diseased tissues. The integration of luminescent probes in in vivo imaging and targeted therapies offers tremendous potential for advancing our understanding of complex biological processes, improving disease diagnosis, and facilitating the development of personalized medicine approaches [217,218,219].

**Figure 18:**
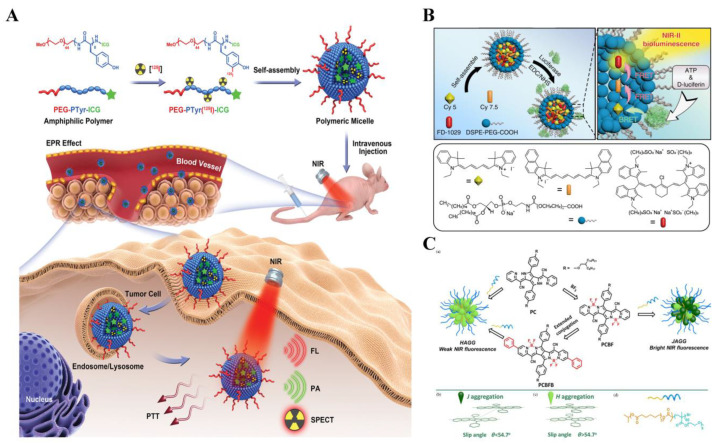
Luminescence probes for imaging and therapy: (**A**) Schematic illustration of the formation of ICG-conjugated and 125I-labeled polymeric micelles for imaging-guided therapy of tumors. Reprinted with permission from ref. [220]. Copyright 2020 Wiley Online Library. (**B**) Concept of NIR-II-BPs with NIR-II bioluminescence. Reprinted with permission from ref. [225]. Copyright 2020 Springer Nature. (**C**) Schematic diagram of encapsulation of the PPcy derivatives into H-type-and J-type-aggregated nanoparticles. (a) The routes to formation of J-aggregate and H-aggregate nanoparticles. (b) Schematic representation of J-aggregate. (c) Schematic representation of H-aggregate. (d) Chemical structure of the amphiphilic block co-polymer. Reprinted with permission from ref. [228]. Copyright 2017 Wiley Online Library.

It was reported that luminescence probes based on heptamethine cyanine dyes, phthalocyanines, and rhodamine analogs were developed for in vivo imaging. Yang et al. strategically prepared polymeric micelles by self-assembling an amphiphilic di-block polymer, which was conjugated with indocyanine green and labeled with radionuclide iodine-125 [220] (Figure 18A). The resulting polymeric micelles demonstrated excellent biocompatibility and passive tumor-targeting ability. Upon tail intravenous injection, the polymeric micelles efficiently accumulated at the tumor site and provided high-sensitivity images with unlimited tissue penetration. Subsequent precision photothermal therapy of tumors was achieved with minimal cumulative side effects upon 808 nm laser irradiation. Yeroslavsky’s study aimed to enhance the stability and efficacy of indocyanine green dye for near-infrared bioimaging and photothermal therapy [221]. By encapsulating ICG and the NIR dye IR-1061 in biocompatible micelles, the reaction between ICG and oxygen was reduced. The micelles exhibited a small size and increased stability, allowing for prolonged heat generation and reduced singlet oxygen (SO) production. The micelles showed potent cytotoxicity against MCF7 cancer cells upon NIR irradiation, with both temperature increase and decomposition debris contributing to their efficacy. In live mice, the micelles exhibited bright fluorescence in the NIR region, enabling visualization of blood vessels and organs.

In addition to molecules and micelles, different forms of nanoparticles, including nanopillar, nanorod, nanostar, and core–shell structure, are also used to carry fluorescent probes [222,223,224]. Lu et al. developed the bioluminescence probes emitting in the second near-infrared region at 1029 nm using bioluminescence resonance energy transfer and two-step FRET with a specially designed cyanine dye FD-1029 [225] (Figure 18B). These biocompatible probes demonstrated enhanced imaging capabilities for vessels and lymphatics in mice, achieving higher signal-to-noise ratios and spatial resolution compared to fluorescence imaging and conventional bioluminescence imaging. The probes also exhibited multiplexed imaging capabilities. Furthermore, due to their ATP-responsive properties, it showed potential for detecting tumor metastasis with a high tumor-to-normal tissue ratio of 83.4.

The limitations of traditional organic fluorophores and probes, tending to diffuse away from the target analytes in biological systems due to their high solubility, pose challenges in their practical application, which results in low signal-to-background ratio and can lead to false positive or negative signals, hindering in situ detection and imaging. Several studies have been reported to address this limitation through self-assembly of organic dyes [226,227,228,229,230,231] (Figure 18C). Yang et al. introduced colloidal nanoparticles that exhibit bright near-infrared fluorescence and narrow bandwidth, making them suitable for bioimaging purposes [228]. The key component is a fluorescent compound called PCBF, which is a derivative of pyrrolopyrrole cyanine, demonstrating emissions in the near-infrared region up to 1000 nm and higher quantum yields compared to other organic near-infrared dyes. The resulting probes exhibit bright NIR fluorescence primarily and demonstrate strong photostability, which is crucial for bioimaging applications. Sun et al. presented a novel approach to develop a new type of the second near-infrared probe. These J-aggregates were formed through the self-assembly of FD-1080 cyanine dyes and DMPC, resulting in absorption and emission properties beyond 1300 nm. The FD-1080 J-aggregates demonstrated excellent hydrophilicity and stability under physiological conditions. Both in vitro and in vivo experiments confirmed the superior imaging capability of the FD-1080 J-aggregates beyond 1500 nm. Additionally, the study employed the second near-infrared imaging to monitor the dynamic changes in the carotid artery of hypertensive rats after the administration of the hypotensor Isoket.

## 4. Conclusions and Perspective

In this review, we provided a comprehensive summary and detailed description of the principles of luminescence probes and recent advances in their bio-applications, in the hope that it will contribute to the readers’ understanding of luminescence probes. We first focused on and concisely described principles of the current widely used and promising luminescence probes, including fluorescence probes, bioluminescence probes, chemiluminescence probes, afterglow probes, photoacoustic probes, and Cerenkov luminescence probes. Considering the practical applications of the probes, we then explored the advances in bio-applications of luminescence probes by category, including metal ions detection, secretion detection, imaging, and therapy. Despite the spectacular progress, the development of luminescence probes for bioanalysis is still on the road. Considerable challenges remain to be overcome to achieve further development, such as the shallow tissue penetration depth, chronic toxicity of many probes containing heavy metal ions, and the relatively weak luminescence signal. To increase the tissue penetration depth, researchers are trying to identify new probes in nature or develop novel probes by genetic engineering and chemical syntheses to reach near-infrared emission, which can bring about excellent tissue penetration depth and bioimaging resolution. More efficient red or near-infrared emitting materials are also a topic worth investigating in the future. Another issue that deserves attention is how to obtain a more effective and persistent luminescence. Although some theoretical progress has been made in the persistent luminescence of single-component systems or hybrid materials, the combination of these into probes or biosensors may be a future research direction. Moreover, to ensure excellent biocompatibility, novel probes that can be cleared rapidly by living organisms should also be developed to reduce their toxic damage to the body. In summary, by highlighting some recent work on luminescence probe principles and bio-applications, we hope to contribute to the development of novel luminescence probes.

## Data Availability

Data are contained within the article.

## Abbreviations

FL	fluorescence
BL	bioluminescence
CL	chemiluminescence
PA	photoacoustic
CT	computed tomography
MRI	magnetic resonance imaging
SBR	signal-to-background
TP	two-photon
CCD	charge-coupled device
FRET	fluorescence resonance energy transfer
BRET	bioluminescence resonance energy transfer
Ln-MOFs	lanthanide metal–organic frameworks
FLIM	fluorescence lifetime imaging
LOD	limit of detection
RSD	relative standard deviations
AFDS	aptamer-functionalized DNA fluorescent sensor
CF	cystic fibrosis
BODIPY	boron-dipyrromethene
QD	quantum dot
ConA	concanavalin A
COPD	chronic obstructive pulmonary disease
Tb	terbium
CP	coordination polymer
NP	nanoparticles
MTX	methotrexate
RCD	red carbon dot
SO	singlet oxygen
NIR	Near infrared
